# Potential of photon-counting detector CT technology for contrast medium reduction in portal venous phase thoracoabdominal CT

**DOI:** 10.1007/s00330-025-11409-3

**Published:** 2025-02-12

**Authors:** Daniel Popp, Martin Siedlecki, Lena Friedrich, Mark Haerting, Christian Scheurig-Muenkler, Florian Schwarz, Thomas Kroencke, Stefanie Bette, Josua A. Decker

**Affiliations:** 1 Department of Diagnostic and Interventional Radiology, University Hospital Augsburg, Faculty of Medicine, University of Augsburg, Augsburg, Germany; 2 Department of Radiology and Nuclear medicine, Kantonspital St. Gallen, St. Gallen, Switzerland; 3 Institute for Radiology, DONAUISAR Hospital Deggendorf-Dingolfing-Landau, Deggendorf, Germany; 4 Medical Faculty, Ludwig Maximilian University Munich, Munich, Germany; 5 Centre for Advanced Analytics and Predictive Sciences (CAAPS), University of Augsburg, Augsburg, Germany

**Keywords:** Photon-counting detector computed tomography, Contrast medium, Iodine contrast, Virtual monoenergetic imaging, Oncologic imaging

## Abstract

**Objectives:**

To compare image quality and iodine attenuation intra-individually in portal venous phase photon-counting detector CT (PCD-CT) scans using protocols with different contrast medium (CM) volume.

**Materials and methods:**

A prospectively acquired patient cohort between 04/2021 and 11/2023 was retrospectively screened if patients had the following combination of portal venous phase thoracoabdominal CT scans: (a) PCD-CT with 120 mL CM volume (PCD-CT_120 mL_), (b) PCD-CT with 100 mL CM volume (PCD-CT_100 mL_), and (c) prior energy-integrating detector CT (EID-CT) with 120 mL CM volume. On PCD-CT, virtual monoenergetic image (VMI) reconstructions at 70 keV were applied for both groups as well as additional VMI at 60 keV for PCD‑CT_100 mL_. Quantitative analyses including signal-to-noise (SNR) and contrast-to-noise ratios (CNR) and qualitative analyses were performed using a mixed linear effects model.

**Results:**

The final study cohort comprised 49 patients (mean age 67 [31–86] years, 12 female). Comparison to EID-CT was available in 33 patients. In standard 70 keV VMI reconstructions, PCD-CT_100 mL_ was non-inferior to PCD-CT_120 mL_ as well as to EID-CT_120 mL_ for CNR in abdominal organs (all *p* > 0.050). The mixed linear effects model revealed significant differences between contrast volume groups for both contrast enhancement and image quality ratings. PCD-CT_100 mL/70 keV_ demonstrated the smallest deviation from optimal contrast enhancement (−0.306, *p* < 0.001).

**Conclusion:**

In portal venous phase thoracoabdominal PCD-CT, a nearly 17% reduction in CM was achievable while maintaining subjective and objective image quality compared to prior higher CM volume PCD-CT scans within the same patients and still surpassing image quality of previous exams on an EID-CT system.

**Key Points:**

***Question*** *How do image quality and iodine attenuation intra-individually compare in portal venous phase photon-counting detector CT (PCD-CT) scans using protocols with different contrast medium volume.*

***Findings*** *PCD-CT scans exhibit superior quantitative and qualitative image quality compared to energy-integrating detector-CT acquisitions and are not negatively affected by contrast volume reductions up to 17%.*

***Clinical relevance*** *This study provides further evidence that PCD-CT enables a considerable reduction in iodine dose for portal venous phase acquisition, benefiting both patients and healthcare system costs.*

## Introduction

Contrast-enhanced thoracoabdominal CT is essential for the work-up of oncologic patients, often leading to high cumulative radiation and contrast medium (CM) doses with potential long-term side effects and associated costs [1, 2]. The administration of iodinated CM must balance diagnostic confidence against risks such as post-contrast acute kidney injury, particularly in cancer patients [3, 4]. In addition, the environmental impact (e.g., groundwater contamination), healthcare costs and the susceptibility to production shortages related to iodine CM are significant concerns [2].

CT in the portal venous phase provides excellent visualization of tumors and lymph nodes in the chest and abdomen, crucial for detecting changes in parenchymal organs (e.g., liver metastases), tumor size, and new lesions, thereby influencing treatment response evaluation [5, 6]. Several factors, such as patient size, radiation dose, but also CM concentration and volume significantly influence image quality [7]. Sufficient iodine dose is crucial for parenchymal contrast enhancement [8], yet CM dosing lacks international standardization [9–11]. Protocols typically use a fixed-dose or a weight-based approach, with 120 or 125 mL being common volumes in fixed-dose protocols [9, 10, 12, 13].

Iodine absorbs low-energy x-ray quanta primarily at its k-edge at 33.2 keV [14, 15]. Unlike conventional energy-integrating detectors (EID), photon-counting detectors (PCD) fully capture and equally weight these low-energy quanta while also minimizing electronic noise, resulting in a higher iodine contrast-to-noise ratio (CNR) with the potential to reduce CM dose [16–18]. Low-energy virtual monoenergetic imaging (VMI) reconstructions further improve CNR and diagnostic confidence, particularly at levels < 70 keV, though they may also increase image noise [17, 19, 20]. Previous studies have shown CM dose reduction in arterial phase PCD-CT, but data for portal venous phase PCD-CT are limited [21–24].

This study aims to compare objective (CNR as a surrogate marker) and subjective (radiologists rating) image quality and contrast enhancement in portal venous phase PCD-CT using 120 mL and 100 mL CM volumes (with additional comparison to 120 mL EID-CT) and to evaluate the potential of 60 keV VMI to compensate for the expected reduction in iodine attenuation.

## Methods

### Study design and patients

This retrospective analysis of a prospectively acquired cohort comprised patients that were scanned between August 2021 and November 2023 (Clinical Trials Registration Number: NCT04989192). The study included consecutive patients who presented to our radiological department for CT of the chest and/or abdomen (either whole or upper abdomen) with known or suspected cancer. The local ethics committee approved this study, and all participants provided written informed consent.

The cohort was screened for patients with the following combination of three contrast-enhanced thoracoabdominal CT scans in portal venous phase: (a) PCD-CT with a CM volume of 120 mL (PCD-CT_120 mL_), (b) PCD-CT with a CM volume of 100 mL (PCD-CT_100 mL_), and (c) EID-CT with a CM volume of 120 mL (EID-CT_120 mL_). Inclusion of PCD-CT_120 mL_ data ended in October 2021, at which time the reference CM dose of the institutional standard protocol for staging PCD-CT was reduced from 120 to 100 mL, which is still in line with current guidelines for contrast agent dosing. The CM volume reduction was made to improve the diagnostic workflow of clinical routine (radiologists of our institution reported a subjective impression of over-enhancement in PCD-CT_120 mL_) and not for study purposes. Regardless of this adjustment, subjects at the upper or lower extremes of the BMI range were excluded to ensure consistency. Specifically, patients with a BMI > 30 kg/m² or < 18 kg/m² were excluded, as their CM volumes would deviate significantly from the standard doses used in this study. BMI (defined as kg/m²) and dose parameters were recorded for all scans.

**Fig. 1:**
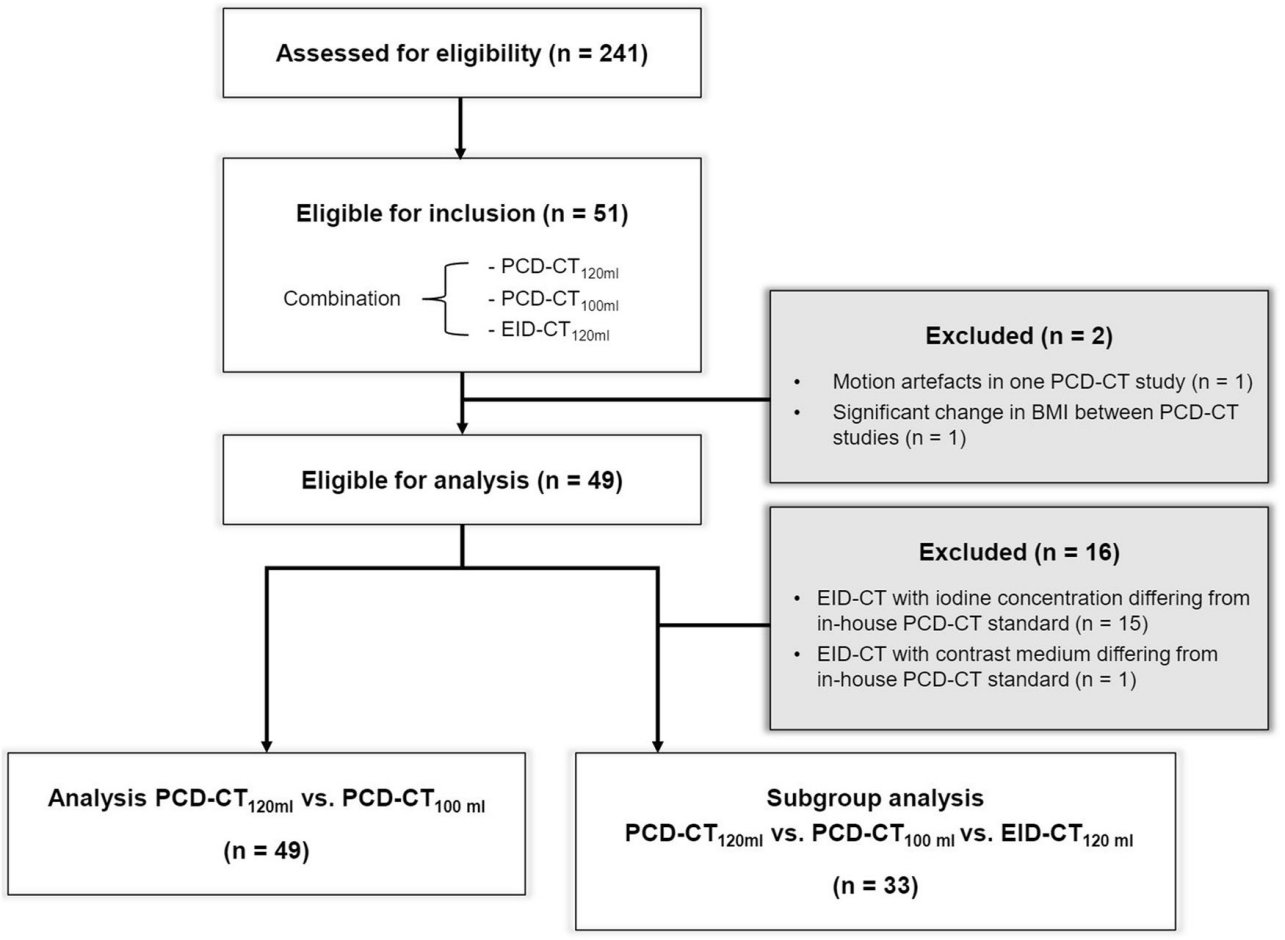
Patient recruitment flowchart

If available, EID-CT scans of the same patients between August 2018 and August 2021 were also included for a retrospective comparison. These EID-CT scans were only included if they matched the criteria of having the same CM volume (120 mL) and were performed within a reasonable time frame (< 36 months) relative to the first PCD-CT scan to ensure comparability. Exclusion criteria comprised age < 18 years, insufficient image quality (considered “not diagnostic” in the radiological report of the study, including motion or metal artifacts and not insufficient contrast), and a significant intra-individual change in BMI between the examinations (> 5 kg/m^2^). Ultimately, 49 patients fulfilled the inclusion criteria for the PCD-CT cohort. Within this cohort, a subset of 33 patients also had prior EID-CT scans available for comparison. An inclusion flowchart is provided in Fig. 1.

### CT image acquisition and reconstruction

For all examinations (PCD- and EID-CT), the scan range was individually defined to cover the region from the upper thoracic aperture to the symphysis (thoracic + full abdominal protocol) or iliac crest (thoracic + upper abdominal protocol), respectively. A forearm vein or port catheter was used to inject a bolus of CM at an iodine concentration of 300 mgI/mL, using either Ultravist 300 (iopromide, Bayer) or Iomeron 300 (iomeprol, Bracco), followed by 30 mL of saline. The flow rate was 4.0 mL/s (forearm vein administration) or 2.5 mL/s (port catheter administration). The scan was triggered with a delay of 45 s when the bolus tracking region of interest in the ascending aorta reached a threshold of 120 HU. Subjects were scanned in a supine position and craniocaudal direction during a single breath-hold.

#### Photon-counting detector CT

All PCD-CT scans were performed using a first-generation PCD-CT system (NAEOTOM Alpha, Siemens Healthineers). Scans prior to February 2022 were performed with a fixed tube voltage of 120 kVp. From February 2022, subjects were scanned with either 120 kVp (*n* = 21) or 140 kVp (*n* = 28) (in the PCD-CT_100 mL_ group), as our department was recommended by the manufacturer to use the scanner-specific tube voltage modulation system (CARE keV, Siemens Healthineers), which automatically selects between predefined tube voltages, depending on the patient’s individual attenuation profile derived from the topogram. Image quality level was either 128 (thoracic + upper abdominal protocol) or 145 (thoracic + full abdominal protocol). Furthermore, the following parameters were applied: 0.25 s rotation time, 0.8 pitch, and 144 × 0.4 mm collimation. Images were obtained using a dedicated acquisition mode with a readout of spectral data (Quantum Plus, Siemens Healthineers; detector-based energy thresholds: 20, 35, 65, and 70 keV). A quantitative soft-tissue kernel with a PCD-CT-specific iterative reconstruction method (QR40, QIR3, Siemens Healthineers) was used to generate SPP (spectral post-processing, Siemens Healthineers) image series – an extended DICOM format containing inherent spectral data. Slice thickness of SPP image series was 1.0 mm with an increment of 0.7 mm.

#### Energy-integrating detector CT

Patients of the comparison group were scanned on one of the following two EID-CT scanners: Somatom Definition AS20 (Siemens Healthineers) or BrightSpeed 16 (GE Healthcare). The relevant scan parameters of both scanners are summarized in Supplementary Table S1.

#### Quantitative CT image analysis

**Fig. 2:**
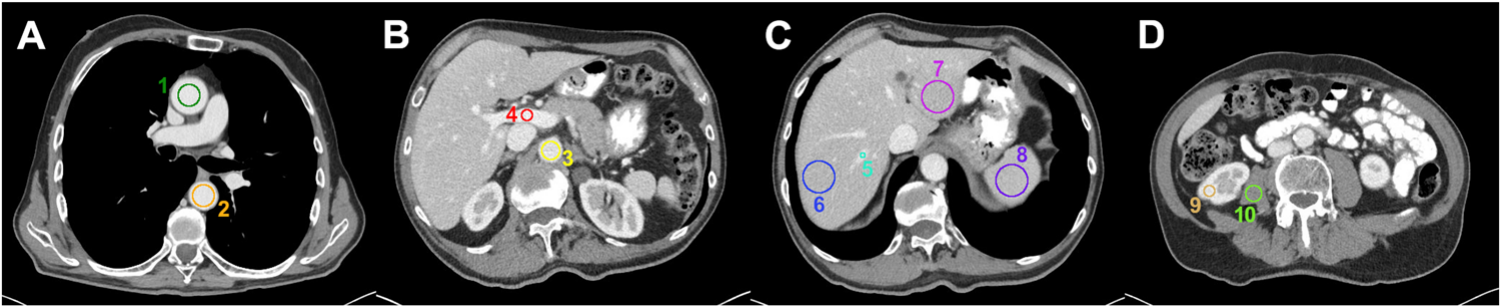
Image slices of a PCD-CT_120 mL/70_ keV dataset (window setting: width 400 HU/level 40 HU) with illustration of the ROI positions for measurements of mean HU and standard deviation of HU: (1) ascending aorta; (2) descending aorta; (3) abdominal aorta; (4) portal vein; (5) liver vein; (6) right liver lobe; (7) left liver lobe; (8) spleen; (9) renal cortex; (10) right psoas muscle

SPP image series of PCD-CT_120 mL_ and PCD-CT_100 mL_, as well as thin-layer EID-CT_120 mL_ image series, were transferred to a workstation with dedicated software (Multimodality Reading, Syngo.via, version VB70A, Siemens Healthineers). In the same soft-tissue window (width 400 HU/ level 40 HU), the following four datasets were displayed and synchronized: (a) PCD-CT_120 mL_, (b) PCD-CT_100 mL_, (c) PCD-CT_100 mL_ at 60 keV (VMI), and (d) EID-CT_120 mL_. Using a multiplanar reconstruction mode, slice thickness was adjusted to 3.0 mm for all datasets. Ten regions of interest (ROIs) were positioned in one dataset and copied to the remaining three datasets using the following locations: (1) ascending aorta at the level of pulmonary bifurcation; (2) descending aorta at the level of pulmonary bifurcation; (3) abdominal aorta at the level of portal vein; (4) portal vein; (5) one liver vein; (6) right liver lobe (segment VI or VII); (7) left liver lobe (segment II or III); (8) spleen; (9) renal cortex (preferably the right kidney); (10) right psoas muscle at the level of the iliac crest (Fig. 2).

Concerning ROIs in vessels, care was taken to avoid atherosclerotic changes and areas of inhomogeneity (contrast-blood mixing). In the liver and spleen, ROIs were positioned in distance to visible vascular and ductal structures, respectively. In the psoas muscle, care was taken to not include intramuscular fat stripes. Means and standard deviations (SD) of Hounsfield units (HU) for each ROI were derived and the following formula were calculated:

$${{\rm{Signal}}}-{{\rm{to}}}-{{\rm{noise\; ratio}}}\!:{SNR}=\quad \frac{{Mea}{n}_{{ROI}}\quad }{{Standard\; Deviatio}{n}_{{ROI}}}$$

$${{\rm{Contrast}}}-{{\rm{to}}}-{{\rm{noise\; ratio}}}\!:{CNR}=\quad \frac{({Mea}{n}_{{ROI}}-{Mea}{n}_{{Psoas}})}{{Standard\; Deviatio}{n}_{{ROI}}}$$

SNR and CNR were calculated as described previously [10, 22].

Subgroup analyses were performed for cases with same kVp settings (kVp = 120) as well as for same kVp (120) and CARE keV settings.

#### Qualitative CT image analysis

**Fig. 3:**
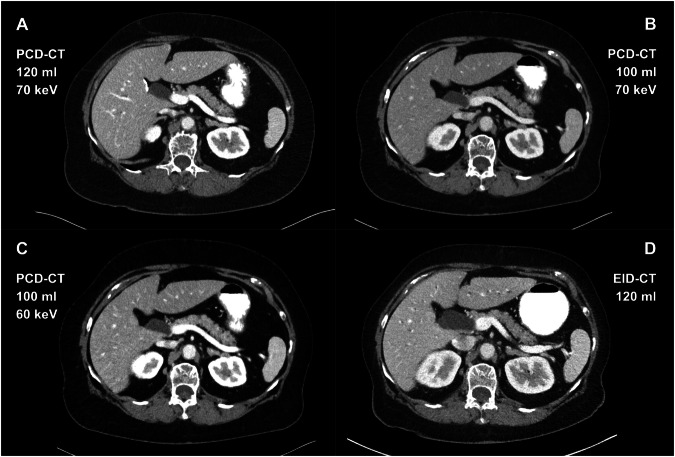
Image slices of three different datasets at the level of the portal vein (same patient, all shown in soft-tissue window: width 400 HU/level 40 HU): PCD-CT_120 mL_ reconstructed in 70 keV (**A**), PCD-CT_100 mL_ reconstructed in 70 keV (**B**) and 60 keV (**C**), and EID-CT_120 mL_ (**D**). Note the slight over-enhancement of the portal vein and kidneys in PCD-CT_120 mL/70 keV_ and PCD-CT_100 mL/60 keV_

Two radiologists with 11 (L.F.) and 4 (M.S.) years of experience in reading thoracoabdominal CT scans independently evaluated overall image quality and overall contrast enhancement of all image studies that had been included for quantitative measurements and were blinded to CM volume, scanner and VMI reconstructions. Image datasets of PCD-CT_120 mL/70 keV_, PCD-CT_100 mL/70 keV_, PCD-CT_100 mL/60 keV_, and, if applicable, EID‑CT_120 mL_ of each case were displayed in the same window and harmonized in terms of slice thickness (3.0 mm) and window setting (width 400 HU/ level 40 HU), respectively (Fig. 3).

Image series were ordered in a random manner, and readers were also blinded to all patient and study information. The rating systems were explained in detail to the readers and no time limit was prescribed.

A five-point Likert scale was utilized to subjectively grade both overall image quality and overall contrast enhancement. The following scales were defined:

Overall image quality: (1) excellent; (2) good; (3) acceptable; (4) poor; (5) very poor.

Overall contrast enhancement: (1) over-enhancement; (2) slight over-enhancement; (3) optimal enhancement; (4) poor enhancement; (5) very poor enhancement.

### Statistical analysis

Statistical analysis was performed using R Statistics (version 4.3.1, R Core Team) [25] and RStudio (version 2023.06.2) [26]. The Shapiro-Wilk test was used for testing of normality distribution. Age and BMI were non-normally distributed. Most CNR / SNR and results of the qualitative assessment were also non-normally distributed, therefore non-parametric tests (Wilcoxon tests for paired samples or Friedman tests with posthoc analysis for more than two groups) were used to compare the different groups. We measured inter-reader agreement using Kendell Tau correlation coefficient [27]. In addition, to account for the clustering effects introduced by individual patient scans and variability between raters, a linear mixed-effects model was applied. The model included the three different PCD-CT datasets as a fixed effect and random intercepts for individual patient scans and raters to control for within-group correlations. For contrast enhancement, the deviation of ratings from the optimal score (3) was modeled to identify significant differences between groups. For image quality, raw scores (1–5) were analyzed directly. The significance of fixed effects was evaluated using *p*-values derived from the model, and pairwise comparisons were conducted to assess intergroup differences.

Spearman correlations were performed to compare quantitative (CNR) and qualitative assessments (Likert scale). Data were analyzed separately for each rater and separately for different CM protocols and regions (e.g., aorta, spleen). Bonferroni correction was applied to correct for multiple testing; we multiplied the observed *p*-value by the number of tests to get the adjusted *p*-value [28]. An adjusted *p*-value ≤ 0.05 was assumed statistically significant. Data visualization was performed using Python (version 3.10). Quantitative data were visualized using boxplots, qualitative data are shown as stacked bar charts.

## Results

### Patient characteristics, scan and dose parameters

Figure 1 visualizes the process of patient recruitment. In total, 241 consecutive patients were screened for eligibility. Of these, 51 subjects had a combination of PCD-CT_120 mL_, PCD-CT_100 mL_, and EID-CT_120 mL_ scans. Two patients were excluded due to motion artefacts in one of the PCD-CT examinations (*n* = 1) and due to significant changes in BMI between the two PCD-CT studies (*n* = 1). The final cohort consisted of 49 patients (median age 68 [31–86] years, 12 female).

For the subgroup analysis with EID-CT, 16 subjects were excluded for having either an EID-CT with an iodine concentration other than 300 mg/mL (*n* = 15) or a different contrast medium from the in-house standard (*n* = 1). Ultimately, 33 patients (6 female) remained for the subgroup analysis. In the comparison of PCD-CT groups (*n* = 49), the median volume computed tomography dose index (CTDI_Vol_) was significantly lower in PCD-CT_100 mL_ compared to PCD-CT_120 mL_ (7.4 [6.1–8.4] vs. 7.7 [6.8–9.3]; *p* < 0.001). In the subgroup, EID-CT had the highest CTDI_Vol_ and PCD-CT_100 mL_ the lowest CTDI_Vol_, with a significant difference between the groups (8.9 [6.2–11.4] vs. 7.3 [6.5–8.3]; *p* = 0.001). All baseline patient characteristics are shown in Table 1 (PCD-CT cohort) and Table 2 (subgroup including EID-CT scans).

**Table 1:** Baseline patient characteristics—PCD-CT groups (*n* = 49)

*n*, female (%)	12/49 (24.5)		
Age, years (median [range])	68.0 [31–86]		
Time frame, days (median [range])	202 [46–772]		
BMI-difference, kg/m² (median [range])	0.7 [−4.1 to 3.1]		
	**PCD-CT 120 mL**	**PCD-CT 100 mL**	***p*-value**
BMI, kg/m² (median [range])	25.3 [18.0–39.2]	24.7 [17.9–39.2]	0.010
CTDI_Vol_, mGy*cm (median [range])	7.7 [3.4–14.5]	7.4 [3.9–13.0]	< 0.001

*BMI* body mass index, *CTDI*_*Vol*_ volume computed tomography dose index, *IQR* interquartile range

**Table 2:** Baseline patient characteristics—subgroup (with EID-CT_120 mL_, *n* = 33)

*n*, female (%)	6/33 (18.2)			
Age, years (mean [range])	68.0 [31–86]			
BMI-difference EID-CT = > PCD-CT 120 mL, kg/m² (median [range])	1.1 [−2.9 to 3.6]			
BMI-difference EID-CT = > PCD-CT 100 mL, kg/m² (median [range])	1.0 [−4.7 to 3.8]			
	**EID-CT**	**PCD-CT 120 mL**	**PCD-CT 100 mL**	*p*-value*
BMI, kg/m² (median [range])	24.5 [21.0–36.8]	25.4 [21.2–39.2]	24.7 [17.9–39.2]	0.151
CTDI_Vol_, mGy*cm (median [range])	8.9 [5.3–18.8]	7.9 [5.6–14.5]	7.3 [4.2–13.1]	0.001
	**EID-CT** = > **PCD-CT 120 mL**	**EID-CT** = > **PCD-CT 100 mL**	**PCD-CT 120 mL = > PCD-CT 100 mL**	
Time difference (median [range], days)	209 [71–1092]	384 [70–1323]	202 [46–772]	< 0.001

*p*-value^1^ = EID-CT vs. PCD-CT 120 mL, *p*-value^2^ = EID-CT vs. PCD-CT 100 mL; *p*-value shown after Bonferroni correction

*BMI* body mass index, *CTDI*_*Vol*_ volume computed tomography dose index, *IQR* interquartile range

* Friedman-test

### Quantitative assessment

Comparison of PCD-CT datasets with 70 keV VMI reconstructions (PCD-CT_120 mL/70 keV_ vs. PCD-CT_100 mL/70 keV_): When reducing the CM volume, there was no significant decrease in SNR across most ROIs (all *p* > 0.050, except the ascending aorta). For CNR, only the descending aorta showed a small but statistically significant decrease.

Comparison of PCD-CT datasets with a CM volume of 100 mL (PCD-CT_100 mL//70 keV_ vs. PCD-CT_100 mL/60 keV_): Compared to 70 keV, there was an increase in CNR across all regions, with statistical significance in seven out of nine regions (except the descending aorta (*p* = 0.073) and spleen (*p* = 0.008)). SNR significantly increased only in the ascending and abdominal aorta, and the portal vein.

**Fig. 4:**
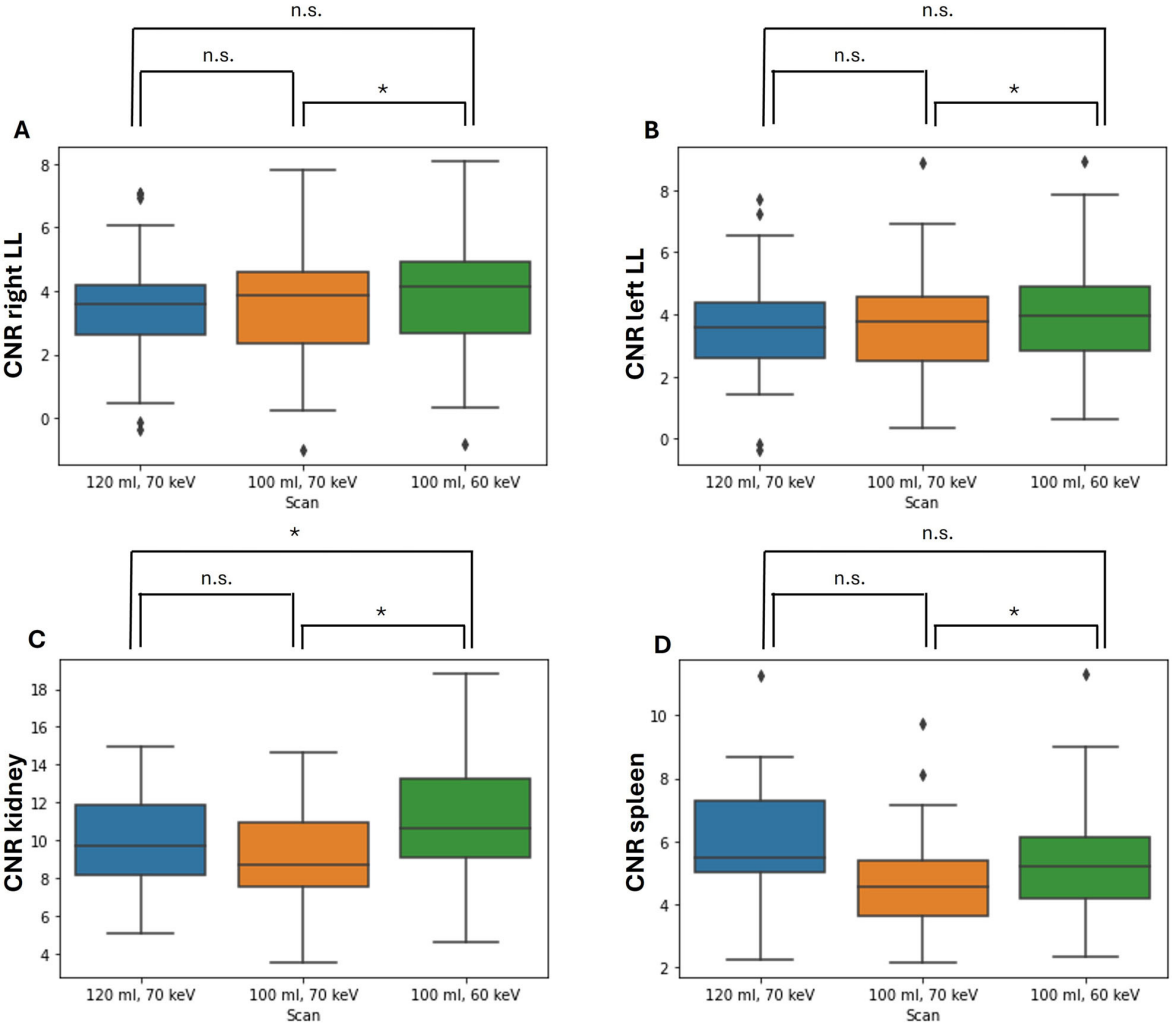
Boxplots for the assessment of abdominal organ CNR (contrast-to-noise ratio)—PCD-CT groups (*n* = 49). LL, liver lobe; * = p < 0.05; n.s., no significant difference

Table 3 provides details of SNR and CNR values with corresponding *p*-values for various locations and comparisons of all PCD-CT examinations (*n* = 49). Boxplots showing a visual comparison of CNR values for the kidney, spleen and liver are presented in Fig. 4.

**Table 3:** Signal-to-noise ratio, Contrast-to-noise ratio—PCD-CT groups (*n* = 49)

	120 mL, 70 keV	100 mL, 70 keV	*p*-value	120 mL, 70 keV	100 mL, 60 keV	*p*-value	100 mL, 70 keV	100 mL, 60 keV	*p*-value
SNR asc. Aorta	17.9 [15.0–20.5]	15.2 [13.3–17.3]	**0.003**	17.9 [15.0–20.5]	16.9 [14.6–18.7]	1.000	15.2 [13.3–17.3]	16.9 [14.6–18.7]	**0.007**
CNR asc. Aorta	12.6 [9.6–14.6]	9.0 [7.5–11.1]	0.059	12.6 [9.6–14.6]	11.2 [9.2–13.4]	1.000	9.0 [7.5–11.1]	11.2 [9.2–13.4]	**0.005**
SNR desc. Aorta	18.5 [14.5–20.4]	15.0 [12.6–16.4]	0.238	18.5 [14.5–20.4]	16.2 [13.3–17.9]	0.025	15.0 [12.6–16.4]	16.2 [13.3–17.9]	0.175
CNR desc. Aorta	12.5 [9.3–14.2]	8.9 [7.2–10.9]	**0.033**	12.5 [9.3–14.2]	10.8 [8.4–12.4]	**0.030**	8.9 [7.2–10.9]	10.8 [8.4–12.4]	0.073
SNR abd. Aorta	10.4 [9.2–13.3]	10.2 [9.1–11.7]	1.000	10.4 [9.2–13.3]	11.6 [9.7–13.2]	1.000	10.2 [9.1–11.7]	11.6 [9.7–13.2]	**0.026**
CNR abd. Aorta	7.8 [5.7–9.0]	6.1 [5.0–7.6]	1.000	7.8 [5.7–9.0]	7.5 [6.5 –9.5]	1.000	6.1 [5.0–7.6]	7.5 [6.5 –9.5]	**0.005**
SNR portal vein	11.8 [10.4–13.5]	12.2 [10.1–14.2]	**0.005**	11.8 [10.4–13.5]	13.7 [11.5–15.4]	**0.002**	12.2 [10.1–14.2]	13.7 [11.5–15.4]	**0.019**
CNR portal vein	8.8 [7.5–10.0]	8.2 [6.6–10.3]	1.000	8.8 [7.5–10.0]	10.3 [8.2–12.1]	0.839	8.2 [6.6–10.3]	10.3 [8.2–12.1]	**0.012**
SNR liver vein	11.9 [10.6–14.6]	13.7 [11.4–17.1]	1.000	11.9 [10.6–14.6]	15.8 [12.2–18.6]	0.149	13.7 [11.4–17.1]	15.8 [12.2–18.6]	1.000
CNR liver vein	8.5 [6.9–10.3]	9.4 [7.4–11.5]	1.000	8.5 [6.9–10.3]	11.8 [8.6–13.9]	**0.007**	9.4 [7.4–11.5]	11.8 [8.6–13.9]	**0.019**
SNR right LL	7.2 [6.2–8.2]	8.2 [6.6–9.6]	1.000	7.2 [6.2–8.2]	8.0 [6.2–9.0]	**0.040**	8.2 [6.6–9.6]	8.0 [6.2–9.0]	0.187
CNR right LL	3.6 [2.6–4.2]	3.9 [2.4–4.6]	1.000	3.6 [2.6–4.2]	4.1 [2.7–4.9]	0.478	3.9 [2.4–4.6]	4.1 [2.7–4.9]	**0.013**
SNR left LL	6.7 [5.7–8.4]	7.7 [6.4–9.4]	**0.005**	6.7 [5.7–8.4]	7.6 [6.2–9.0]	0.231	7.7 [6.4–9.4]	7.6 [6.2–9.0]	0.191
CNR left LL	3.6 [2.6–4.4]	3.8 [2.5–4.5]	1.000	3.6 [2.6–4.4]	3.9 [2.8–4.9]	0.296	3.8 [2.5–4.5]	3.9 [2.8–4.9]	**0.048**
SNR spleen	9.2 [7.9–10.9]	9.4 [8.1–10.2]	1.000	9.2 [7.9–10.9]	9.4 [7.8–10.3]	1.000	9.4 [8.1–10.2]	9.4 [7.8–10.3]	1.000
CNR spleen	5.5 [5.0–7.3]	4.6 [3.6–5.4]	0.378	5.5 [5.0–7.3]	5.2 [4.2–6.1]	1.000	4.6 [3.6–5.4]	5.2 [4.2–6.1]	0.008
SNR kidney	12.8 [11.3–15.9]	13.4 [11.6–16.0]	0.881	12.8 [11.3–15.9]	14.1 [12.6–17.2]	**0.003**	13.4 [11.6–16.0]	14.1 [12.6–17.2]	0.056
CNR kidney	9.7 [8.2–11.8]	8.7 [7.5–10.9]	1.000	9.7 [8.2–11.8]	10.6 [9.1–13.3]	**0.011**	8.7 [7.5–10.9]	10.6 [9.1–13.3]	**0.005**

Data shown as median [interquartile range]. Where differences between groups were statistically significant, p-values are shown in bold

*SNR* signal-to-noise ratio, *CNR* contrast-to-noise ratio, *LL* liver lobe

#### Subgroup analysis comparing with EID-CT reference scans

In the subgroup analysis of patients with available EID-CT reference scan (*n* = 33), CNR was lowest in EID-CT_120 mL_ and remained below the levels observed in the three PCD-CT groups in almost all locations (except the ascending aorta and the left liver lobe). SNR was lowest in EID-CT across all regions compared to PCD-CT. Supplementary Table S2 shows the median SNR and CNR with corresponding *p*-values for the various locations of all scans included in the subgroup analysis (*n* = 33). Boxplots of abdominal organ CNR are shown in Supplementary Fig. S1.

*Subgroup analysis comparing cases with same kVp settings (kVp = 120)*

To account for changes in kVp over time, we performed a subgroup analysis including only cases with same kVp (120). For PCD-CT cases, this results in a subgroup of 21 patients. No significant differences were observed between PCD-CT_120 mL_ and PCD-CT_100 mL/70 keV_ in almost all measured regions (*p* > 0.050 except for CNR of the descending aorta) (Table 4).

**Table 4:** Signal-to-noise ratio, contrast-to-noise ratio—PCD-CT groups with same kVp (*n* = 21)

	120 mL, 70 keV	100 mL, 70 keV	*p*-value	120 mL, 70 keV	100 mL, 60 keV	*p*-value	100 mL, 70 keV	100 mL, 60 keV	*p*-value
SNR asc. Aorta	19.3 (15.0–20.6)	15.5 (14.1–17.5)	0.756	19.3 (15.0–20.6)	16.9 (15.1–20.3)	1.000	15.5 (14.1–17.5)	16.9 (15.1–20.3)	**0.003**
CNR asc. Aorta	13.3 (10.2–15.3)	9.2 (7.4–11.8)	0.221	13.3 (10.2–15.3)	11.3 (9.3–13.4)	1.000	9.2 (7.4–11.8)	11.3 (9.3–13.4)	**0.004**
SNR desc. Aorta	19.0 (15.2.21.6)	15.4 (13.5–16.9)	0.103	19.0 (15.2.21.6)	16.4 (14.3–17.9)	1.000	15.4 (13.5–16.9)	16.4 (14.3–17.9)	0.093
CNR desc. Aorta	13.5 (10.4–16.3)	8.9 (7.5–11.6)	**0.046**	13.5 (10.4–16.3)	11.0 (8.9–12.5)	1.000	8.9 (7.5–11.6)	11.0 (8.9–12.5)	**0.004**
SNR abd. Aorta	11.1 (10.0–12.4)	9.6 (8.2–11.8)	1.000	11.1 (10.0–12.4)	10.7 (8.4–13.1)	1.000	9.6 (8.2–11.8)	10.7 (8.4–13.1)	**0.014**
CNR abd. Aorta	7.9 (6.9–8.8)	5.7 (4.9–7.8)	0.178	7.9 (6.9–8.8)	6.9 (5.7–9.3)	1.000	5.7 (4.9–7.8)	6.9 (5.7–9.3)	**0.004**
SNR portal vein	11.8 (10.8–13.1)	11.0 (9.9–14.1)	1.000	11.8 (10.8–13.1)	12.1 (11.3–15.2)	1.000	11.0 (9.9–14.1)	12.1 (11.3–15.2)	0.087
CNR portal vein	8.7 (7.7–10.1)	7.1 (6.1–11.1)	1.000	8.7 (7.7–10.1)	9.0 (7.7–12.1)	1.000	7.1 (6.1–11.1)	9.0 (7.7–12.1)	**0.013**
SNR liver vein	11.8 (11.0–13.1)	12.7 (10.7–14.3)	1.000	11.8 (11.0–13.1)	14.0 (11.9–17.0)	0.624	12.7 (10.7–14.3)	14.0 (11.9–17.0)	**0.009**
CNR liver vein	8.5 (7.7–9.3)	8.9 (6.7–10.2)	1.000	8.5 (7.7–9.3)	10.4 (8.4–12.5)	0.694	8.9 (6.7–10.2)	10.4 (8.4–12.5)	**0.008**
SNR right LL	6.8 (5.5–7.9)	7.0 (6.1–8.3)	0.720	6.8 (5.5–7.9)	6.8 (5.8–8.1)	1.000	7.0 (6.1–8.3)	6.8 (5.8–8.1)	0.057
CNR right LL	3.4 (2.3–4.1)	3.3 (2.2–4.1)	1.000	3.4 (2.3–4.1)	3.5 (2.4–4.5)	1.000	3.3 (2.2–4.1)	3.5 (2.4–4.5)	**0.005**
SNR left LL	6.6 (5.7–7.4)	6.6 (5.8–9.1)	0.823	6.6 (5.7–7.4)	6.5 (5.4–8.7)	1.000	6.6 (5.8–9.1)	6.5 (5.4–8.7)	**0.011**
CNR left LL	3.3 (2.7–4.1)	3.5 (2.2–4.5)	1.000	3.3 (2.7–4.1)	3.5 (2.2–4.8)	1.000	3.5 (2.2–4.5)	3.5 (2.2–4.8)	**0.023**
SNR spleen	9.7 (7.9–11.3)	9.0 (7.4–10.1)	1.000	9.7 (7.9–11.3)	9.0 (7.6–10.1)	1.000	9.0 (7.4–10.1)	9.0 (7.6–10.1)	1.000
CNR spleen	5.8 (5.2–7.5)	4.6 (3.6–5.5)	0.078	5.8 (5.2–7.5)	5.1 (4.2–6.0)	0.559	4.6 (3.6–5.5)	5.1 (4.2–6.0)	**0.004**
SNR kidney	13.1 (11.6–16.4)	12.8 (11.3–14.6)	1.000	13.1 (11.6–16.4)	13.1 (12.2–16.4)	1.000	12.8 (11.3–14.6)	13.1 (12.2–16.4)	0.311
CNR kidney	9.8 (8.2–12.3)	8.6 (7.4–10.3)	0.227	9.8 (8.2–12.3)	9.9 (8.7–12.3)	1.000	8.6 (7.4–10.3)	9.9 (8.7–12.3)	**0.002**

Data shown as median [interquartile range]. Where differences between groups were statistically significant, p-values are shown in bold

*p*-value shown after Bonferroni correction

*SNR* signal-to-noise ratio, *CNR* contrast-to-noise ratio, *LL* liver lobe

Another subgroup analysis included only patients with same kVp (= 120) and same Care keV image quality level (= 128), resulting in a total of 11 patients. Data is shown in Supplementary Table S3. A contrast reduction from 120 mL to 100 mL did not significantly affect SNR and CNR in all measured areas (all *p* > 0.050).

For cases including EID-CT reference scans, the subgroup consists of six patients. The subgroups did not differ significantly (all *p* = 1.000) (Supplementary Table S4).

### Qualitative assessment

Using a linear mixed-effects model, we evaluated the impact of contrast volume/keV settings on both image quality and contrast enhancement ratings while accounting for clustering effects of patient scans and raters. For contrast enhancement, PCD-CT_100 mL/70 keV_ demonstrated the smallest deviation from optimal contrast ratings 3 (difference: 0.306, *p* < 0.001), significantly outperforming PCD-CT_120 mL/70 keV_ (difference: 0.612, *p* < 0.001) and PCD-CT_100 mL/60 keV_ (reference group, intercept: 2.326).

For image quality, PCD-CT_120 mL/70 keV_ demonstrated the smallest deviation from the optimal rating of 1 (difference: −0.286, *p* < 0.001), while PCD-CT_100 mL/60 keV_, as the reference group, had a mean rating of 1.282 (intercept). PCD-CT_100 mL/70 keV_ had a slightly larger deviation from the optimal rating (difference: −0.357, *p* < 0.001). Random effects analysis showed minimal variability attributable to patients for image quality (variance = 0.005) and moderate variability for contrast enhancement (variance = 0.051). Variability between raters was moderate for both contrast enhancement (variance = 0.265) and for image quality (variance = 0.348). In addition, Table 5 provides pairwise differences and comparisons of each group.

**Table 5:** Qualitative assessment—comparison between different groups

Comparison group 1	Comparison group 2	Contrast mean difference	*p*-value	Adjusted *p*-value	Image quality mean difference	*p*-value	Adjusted *p*-value
PCD-CT_100 mL/60 keV_	PCD-CT_100 mL/70 keV_	0.612	< 0.001	< 0.001	−0.286	< 0.001	< 0.001
PCD-CT_100 mL/60 keV_	PCD-CT_120 mL/70 keV_	0.306	< 0.001	< 0.001	−0.357	< 0.001	< 0.001
PCD-CT_100 mL/70 keV_	PCD-CT_120 mL/70 keV_	−0.306	< 0.001	< 0.001	−0.071	0.061	0.182

**Fig. 5:**
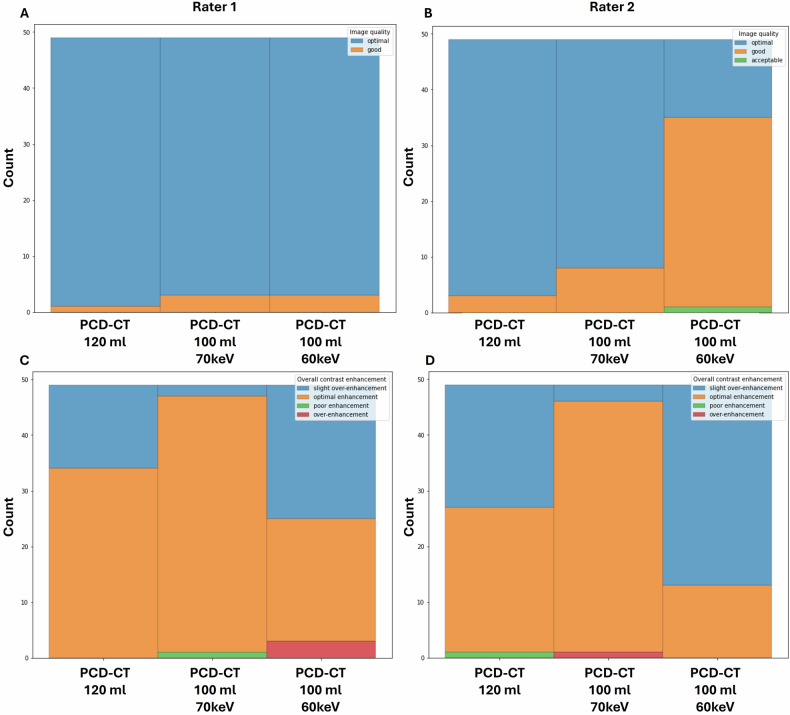
Stacked bar charts showing the distribution of ratings given for overall image quality and overall contrast enhancement for the different PCD-CT groups (*n* = 49)

Comparing both readers in the different groups, we observed moderate to excellent agreement (for example, *r* = 0.550 for assessment of contrast enhancement for PCD-CT_120 mL_ and *r* = 0.348 for assessment of image quality for PCD-CT_100 mL/70 keV_). Figure 5 shows the distribution of subjective scores given for overall image quality and contrast enhancement (iodine attenuation) between the different PCD-CT groups (*n* = 49). Data are presented separately for both raters.

#### Rater 1

Overall image quality: PCD-CT_120 mL/70 keV_ was rated best (98% score 1 = optimal; 2% score 2 = good), followed closely by both PCD-CT_100 mL/70 keV_ and PCD-CT_100 mL/60 keV_ (both: 93.8% score 1; 6.2% score 2).

Contrast enhancement: PCD-CT_100 mL/70 keV_ was rated best for contrast enhancement (93.8% score 3 = optimal enhancement, 4.1% score 2 = slight over-enhancement, 2% score 4 = poor enhancement). A tendency towards slight over-enhancement was seen in PCD-CT_120 mL/70 keV_ (69.4% score 3; 30.6% score 2) and more pronounced in PCD-CT_100 mL/60 keV_ (46.9% score 2; 44.9% score 3, 6.1%; score 1 = over-enhancement).

#### Rater 2

Overall image quality: PCD-CT_120 mL/70 keV_ was rated best (93.8% score 1 = optimal; 6.2% score 2 = good), followed closely by PCD-CT_100 mL/70 keV_ (83.7% score 1; 16.3% score 2). PCD-CT_100 mL/60 keV_ received the lowest score for image quality (30.6% score 1; 67.4% score 2, 2% score 3 = acceptable).

Contrast enhancement: PCD-CT_100 mL/70 keV_ was rated best for contrast enhancement (91.9% score 3 = optimal enhancement, 6.1%; score 2 = slight over-enhancement, 2% score 1: over-enhancement). A tendency towards slight over-enhancement was also observed in PCD-CT_120 mL/70 keV_ (55.1% score 3; 44.9% score 2) and more pronounced in PCD-CT_100 mL/60 keV_ (73.5% score 2; 26.5% score 3).

In the subgroup analysis (*n* = 33), the ranking of PCD groups was comparable to the larger cohort analysis (PCD-CT only), both in terms of image quality and contrast enhancement. EID-CT_120 mL_ was graded lowest (Rater 1) together with PCD-CT_100 mL/60 keV_ for image quality (Rater 2), but EID-CT received the best score for contrast enhancement together with PCD-CT_100 mL/70 keV_. For illustration, see Supplementary Fig. 2.

#### Comparison between quantitative and qualitative assessment

Spearman correlations were performed to test objective enhancement and subjective perception of contrast enhancement. Data are presented in Supplementary Table S5. In summary, we observed negative correlations between CNR and qualitative assessment; accordingly, higher CNR was more likely to be rated as over-enhancement.

## Discussion

This study evaluated the potential for reducing contrast medium (CM) in portal venous phase PCD-CT scans by comparing 120 mL and 100 mL CM volumes, conducting a subgroup analysis with additional 120 mL EID-CT reference scans, and assessing the use of 60 keV VMI reconstructions to compensate for reduced iodine contrast. The main results of this study were as follows: (a) In PCD-CT, a reduction of the CM volume of about 17% is feasible while maintaining overall image quality; (b) subjective contrast enhancement of the reduced PCD-CT_100 mL/70 keV_ was non-inferior to 120 mL EID-CT or PCD-CT scans; (c) CNR of parenchymal abdominal organs can be significantly increased by using 60 keV VMI with the downside of an increasing subjective over-enhancement (d) PCD-CT scans generally exhibited superior quantitative (SNR and CNR) image quality at lower radiation dose compared to EID-CT.

Our study aimed to fill the current knowledge gap regarding the potential for CM reduction in portal venous phase PCD-CT, with a particular focus on the contrast enhancement of parenchymal abdominal organs. In contrast to CT angiography, which can be achieved with relatively small doses of precisely timed CM to visualize arteries, effective parenchymal organ imaging typically necessitates the administration of larger CM doses [29, 30]. Presently, however, there are no universally accepted guidelines for the iodine dose necessary to achieve optimal enhancement in portal venous phase PCD-CT, such as the oncological staging CTs in our study. Incorporating the findings from previous studies and our initial experience with our PCD-CT scanner, we assumed that a CM volume reduction by approximately 17% (based on our in-house standard for reference EID-CT = 120 mL) appears achievable. We were able to confirm this assumption by demonstrating that the CNR of nearly all examined regions did not significantly differ between the two PCD-CT groups (using VMI reconstructions at a given keV level). A recent study by Hagen et al examined the potential of reducing CM volume in portal venous phase PCD-CT datasets [30]. The authors compared abdominal PCD-CT to dual source EID-CT scans using a standardized exam protocol, all reconstructed as polychromatic images and as additional VMI at two different keV levels (40 and 70 keV). Comparable to our results, they demonstrated that the image quality remained consistent when reducing the CM volume by 27%. However, a direct comparison to our study remains difficult since Hagen et al utilized weight-adapted protocols and a cohort consisting solely of overweight and obese individuals [30, 31].

Several clinical studies have examined the effect of reducing iodine dose on image quality in PCD-CT scans acquired in arterial phase, including angiography of the pulmonary arteries [22, 32], aorta [21], and coronary arteries [24]. The results of these studies suggest that PCD-CT angiography with substantially reduced CM dose can maintain diagnostic image quality, particularly when utilizing low-keV VMI reconstructions. [14, 16]. However, a direct comparison to these studies is difficult due to the different protocols (in particular, different acquisition phase and higher CM doses in our study).

Based on current research and our own experience, VMI reconstructions at 60 keV provide a good trade-off between an increased delineation of liver lesions in VMI at lower-keV levels, while at the same time only slightly increasing noise [19, 20, 33, 34]. This is the reason why the standard protocol for staging PCD-CT at our institute includes 60 keV VMI reconstructions in addition to the 70 keV ones, and why we have therefore included both in the PCD_100 mL_ group in this study. As expected, CNR in PCD_100 mL_ increased in all analyzed regions when the keV level was lowered. In the subjective analysis, however, this increase in CNR was associated with suboptimal scores for overall enhancement, indicating a tendency towards slight over-enhancement. Besides, a similar tendency was also observed in the PCD_120 mL/70 keV_ group. This can be explained by the fact that in these two groups, the iodine attenuation, particularly of the portal vein and renal cortex, was perceived as too high by both readers, thus necessitating a subsequent adjustment of the windowing settings for a better assessment (see also Fig. 3). These subjective findings also aligned with the CNR values of the portal vein and kidney, which were higher in both PCD_120 mL/70 keV_ and PCD_100 mL/60 keV_ compared to PCD_100 mL/70 keV_. Subjective image quality was rated lowest in PCD_100 mL/60 keV_ (together with EID-CT), most likely due to the nature of lower-keV VMI, as they are known to increase image noise [35, 36]. Thus, when considering the subjective image quality and contrast enhancement together, one can conclude that in our study, PCD-CT_100 mL/70 keV_ represents the best compromise between both.

Image quality is influenced by various variables. First, the use of iterative reconstruction software is capable of significantly enhancing image quality by reducing noise and therefore improving SNR. The specific quantum iterative reconstruction method (QIR3) used in the PCD-CT scans likely additionally contributed to the superior CNR and SNR values compared to EID-CT [37]. Second, different VMI settings also influenced image quality. Lowering VMI-levels increases iodine contrast but also image noise [38, 39]. 70 keV VMI provided a good balance between contrast enhancement and noise, while 60 keV VMI increased CNR but led to higher subjective ratings of over-enhancement, indicating the need for careful VMI selection. Third, although some studies [40, 41] used polychromatic T3D for comparison with EID-CT, we chose 70 keV VMI for its consistency with traditional polychromatic images and clinical relevance (70 keV VMI is the diagnostic reference in our department).

To the best of our knowledge, our study is one of the first that investigated image quality and contrast enhancement in clinical portal venous phase PCD-CT directly compared to reference EID-CT. As explained above, PCD-CT-derived VMI at 70 keV mimic conventional polychromatic images from EID-CT and were therefore used for this comparison. Our analysis revealed higher SNR and CNR values in almost all regions in PCD-CT_120/70 keV_ compared to EID-CT_120 mL_. This aligns with the previous study of Wrazidlo et al on oncological patients, which also found higher SNR and CNR in both vessels and parenchymatous abdominal organs in portal venous polychromatic PCD-CT scans (QIR3 reconstructions) compared to reference EID-CT [42].

Our study has several limitations: The first limitation of this study is its relatively small sample size. Second, we did not assess the diagnostic accuracy of the PCD_100 mL_ protocol, and therefore cannot provide evidence for its non-inferiority compared to the standard protocol, for example, in terms of detectability of liver lesions. However, all applied contrast doses used were within the range of current societal guidelines [43] and all scans primarily included in the prospective study cohort were considered diagnostic by the attending radiologist. Third, there was a slight but significant difference in the median BMI between the PCD_120 mL_ and PCD_100 mL_ group (25.3 vs. 24.7; *p* = 0.01), which means that it cannot be entirely ruled out that the better overall performance of PCD_100 mL_ might be partly explained by the slightly lower BMI values. Fourth, based on the manufacturer’s recommendation, CARE keV (Siemens Healthineers) was included in our thoracoabdominal PCD-CT protocol as of February 2022 (during the time of patient inclusion). This resulted in 28 subjects from the PCD-CT_100 mL_ group being scanned with a different tube voltage (140 kVp) than the remaining patients in this group and those of the PCD-CT_120 mL_ group (all 120 kVp). Nevertheless, we believe that this methodological difference did not significantly influence the overall conclusion (non-inferiority of PCD-CT_100 mL_), as it has already been demonstrated that CNR further decreases when increasing the tube voltage from 120 to 140 kVp, and only an increase could have altered the outcome in the opposite direction [44, 45]. And also, the subgroup analyses from this study, including patients with same 120 kVp only, did show similar results and confirmed the non-inferiority of a contrast reduction from 120 mL to 100 mL. Fifth, while all images were read in a single session, we recognize that the distinct image characteristics of PCD-CT and older EID-CT systems could potentially be identified by experienced radiologists, introducing a bias that may not have been fully accounted for in our analysis. Finally, the different patient groups were not homogenous in terms of radiation dose with PCD-CT_100 mL_ exhibiting a significantly lower median CTDI_Vol_ as compared to PCD-CT_120 mL_ and EID-CT_120 mL_. When comparing only the two PCD-CT groups, this difference is relatively surprising considering the lower average tube voltages applied in the PCD-CT_120 mL_ group. However, this could be explained by the slightly higher average BMI in this group, as higher BMI values are known to be associated with higher radiation doses [46–48].

## Conclusion

This study demonstrates the feasibility of a nearly 17% CM dose reduction in portal venous phase thoracoabdominal PCD-CT while maintaining image quality compared to standard-dose PCD-CT acquisitions and even surpassing reference EID-CT acquisitions. VMI reconstructions at 60 keV can be used to enhance iodine contrast of parenchymal abdominal organs in reduced CM scans, with the potential disadvantage of an overall subjective over-enhancement.

## Supplementary information

Electronic Supplementary Material

## Abbreviations

CM: Contrast medium
CNR: Contrast-to-noise ratio
CTDI_Vol_: Volume computed tomography dose index
EID-CT: Energy-integrating detector computed tomography
PCD-CT: Photon-counting detector computed tomography
ROI: Region of interest
SNR: Signal-to-noise ratio
SPP: Spectral post processing
VMI: Virtual monoenergetic imaging
