# Supplementary material for: Potential of photon-counting detector CT technology for contrast medium reduction in portal venous phase thoracoabdominal CT
Source: Eur Radiol. 2025 Feb 12;35(8):4635–48. doi: 10.1007/s00330-025-11409-3 (PMC12226673; doi:10.1007/s00330-025-11409-3)

Potential of Photon-Counting Detector CT Technology for Contrast Medium Reduction in Portal Venous Phase Thoracoabdominal CT

ELECTRONIC SUPPLEMENTARY MATERIAL

Supplementary Table S1 EID-CT scan parameters

	**Siemens Somatom Definition AS20**	**GE BrightSpeed 16**
n	19	14
Scanner type	20-slice MD-CT	16-slice MD-CT
Tube voltage	100 kVp	120 kVp
Rotation time	0.5 s	0.6 s
Pitch factor	1.05	1.375
Collimation	16 x 1.2 mm	16 x 1.25
Reference mAs	161	250
Automatic exposure control	CARE Dose4D	/
Convolutional kernel	I31f	SOFT
Iterative reconstruction mode	SAFIRE, level 3	/
Slice thickness	1.5	1.25
Increment	1.2	0.625
CARE Dose 4D adaption strengths	7 /10	n.a.

Supplementary Table S2 Signal-to-noise ratio, Contrast-to-noise ratio - subgroup (with EID-CT_{120ml}, n = 33)

	**EID-CT, 120 ml**	**PCD-CT 120 ml, 70 keV**	**P-Value**	**EID-CT, 120 ml**	**PCD-CT 100 ml, 70 keV**	**P-Value**	**EID-CT, 120 ml**	**PCD-CT 100 ml, 60 keV**	**P-Value**
SNR asc. Aorta	13.9 [12.2-17.6]	17.8 [14.5-20.3]	**0.016**	13.9 [12.2-17.6]	15.2 [13.3-17.3]	1.000	13.9 [12.2-17.6]	16.9 [14.5-18.5]	0.126
CNR asc. Aorta	10.4 [7.5-13.0]	12.0 [9.2-14.2]	0.088	10.4 [7.5-13.0]	9.0 [7.6-11.1]	1.000	10.4 [7.5-13.0]	11.7 [9.2-13.3]	0.574
SNR desc. Aorta	12.4 [9.5-14.3]	17.4 [14.3-20.5]	0.301	12.4 [9.5-14.3]	15.0 [12.7-16.7]	0.093	12.4 [9.5-14.3]	16.2 [13.4-17.9]	0.010
CNR desc. Aorta	8.5 [5.6-9.7]	12.8 [8.7-14.9]	0.630	8.5 [5.6-9.7]	9.0 [7.1-11.0]	1.000	8.5 [5.6-9.7]	10.9 [8.4-12.6]	**0.014**
SNR abd. Aorta	8.2 [6.7-10.2]	10.3 [9.2-11.9]	0.760	8.2 [6.7-10.2]	10.1 [9.1-11.7]	**0.004**	8.2 [6.7-10.2]	11.6 [9.6-13.2]	0.151
CNR abd. Aorta	5.1 [4.0-6.6]	7.8 [5.5-8.8]	**0.002**	5.1 [4.0-6.6]	6.3 [5.0-7.6]	0.324	5.1 [4.0-6.6]	8.0 [6.5-9.4]	0.165
SNR portal vein	10.0 [8.5-12.3]	11.9 [10.1-13.7]	**0.003**	10.0 [8.5-12.3]	12.3 [10.5-14.2]	**0.013**	10.0 [8.5-12.3]	13.7 [11.4-15.6]	0.366
CNR portal vein	6.9 [5.7-8.9]	9.3 [7.5-10.4]	**0.013**	6.9 [5.7-8.9]	8.4 [7.1-10.4]	0.495	6.9 [5.7-8.9]	10.6 [8.4-12.1]	0.279
SNR liver veins	10.3 [9.0-12.2]	11.9 [10.8-13.7]	0.107	10.3 [9.0-12.2]	13.3 [11.8-16.2]	**0.004**	10.3 [9.0-12.2]	16.0 [12.5-18.0]	0.196
CNR liver veins	6.8 [5.8-8.9]	8.2 [7.1-9.7]	0.631	6.8 [5.8-8.9]	9.5 [7.8-11.2]	0.053	6.8 [5.8-8.9]	12.2 [9.3-13.6]	0.312
SNR right LL	6.2 [4.9-6.6]	6.9 [5.9-7.7]	0.279	6.2 [4.9-6.6]	7.9 [6.6-9.4]	0.128	6.2 [4.9-6.6]	7.5 [6.2-9.1]	0.153
CNR right LL	2.9 [2.2-3.5]	3.3 [2.5-4.1]	1.000	2.9 [2.2-3.5]	3.6 [2.6-4.6]	0.279	2.9 [2.2-3.5]	3.8 [2.7-5.1]	**0.011**
SNR left LL	6.3 [5.3-7.3]	6.6 [5.5-8.1]	1.000	6.3 [5.3-7.3]	7.7 [6.4-9.0]	0.842	6.3 [5.3-7.3]	7.5 [6.3-8.7]	**0.026**
CNR left LL	3.2 [2.6-4.0]	3.0 [2.5-4.4]	1.000	3.2 [2.6-4.0]	3.7 [2.7-4.4]	1.000	3.2 [2.6-4.0]	3.9 [2.9-4.8]	0.362
SNR spleen	6.8 [5.9-8.3]	9.2 [8.0-11.5]	0.125	6.8 [5.9-8.3]	9.3 [8.1-10. 1]	0.336	6.8 [5.9-8.3]	9.1 [7.9-10.2]	0.258
CNR spleen	3.7 [3.1-5.0]	5.8 [5.0-7.7]	0.232	3.7 [3.1-5.0]	4.8 [3.8-5.5]	**0.042**	3.7 [3.1-5.0]	5.2 [4.4-6.5]	0.534
SNR kidney	9.8 [8.1-11.4]	12.9 [11.3-16.0]	0.151	9.8 [8.1-11.4]	13.4 [11.6-15.6]	0.215	9.8 [8.1-11.4]	14.0 [12.8-17.0]	0.197
CNR kidney	6.8 [5.5-7.8]	9.7 [8.4-12.1]	0.109	6.8 [5.5-7.8]	8.9 [7.3-10.9]	**0.003**	6.8 [5.5-7.8]	10.5 [9.2-13.1]	0.132

Data shown as median [interquartile range], SNR = signal-to-noise ratio, CNR = contrast-to-noise ratio, LL = liver lobe

Supplementary Table 3 Signal-to-noise ratio, Contrast-to-noise ratio – PCD-CT groups with same kVp and same Care keV image quality level (128) (n = 11)

	120 ml, 70 keV	100 ml, 70 keV	P-Value	120 ml, 70 keV	100 ml, 60 keV	P-Value	100 ml, 70 keV	100 ml, 60 keV	P-Value
SNR asc. Aorta	19.3 (16.8-22.3)	16.9 (14.3-20.2)	1.000	19.3 (16.8-22.3)	16.9 (15.7-22.1)	1.000	16.9 (14.3-20.2)	16.9 (15.7-22.1)	0.052
CNR asc. Aorta	13.9 (10.6-17.0)	9.3 (7.6-14.8)	1.000	13.9 (10.6-17.0)	11.1 (9.4-16.5)	1.000	9.3 (7.6-14.8)	11.1 (9.4-16.5)	0.141
SNR desc. Aorta	17.6 (15.2-21.8)	15.4 (13.5-17.4)	0.176	17.6 (15.2-21.8)	16.3 (14.2-18.0)	1.000	15.4 (13.5-17.4)	16.3 (14.2-18.0)	1.000
CNR desc. Aorta	13.1 (10.3-16.4)	8.9 (7.4-11.9)	1.000	13.1 (10.3-16.4)	10.9 (8.8-12.8)	1.000	8.9 (7.4-11.9)	10.9 (8.8-12.8)	0.352
SNR abd. Aorta	10.2 (9.8-11.9)	11.6 (8.2-12.3)	1.000	10.2 (9.8-11.9)	12.5 (8.8-13.3)	0.667	11.6 (8.2-12.3)	12.5 (8.8-13.3)	0.035
CNR abd. Aorta	7.9 (6.5-8.8)	6.3 (4.6-9.2)	1.000	7.9 (6.5-8.8)	8.0 (5.7-11.0)	1.000	6.3 (4.6-9.2)	8.0 (5.7-11.0)	0.141
SNR portal vein	11.6 (10.5-12.3)	10.7 (9.6-13.2)	1.000	11.6 (10.5-12.3)	12.4 (10.7-15.2)	0.879	10.7 (9.6-13.2)	12.4 (10.7-15.2)	0.070
CNR portal vein	8.9 (7.9-9.7)	7.6 (6.3-10.4)	1.000	8.9 (7.9-9.7)	10.2 (7.3-12.4)	1.000	7.6 (6.3-10.4)	10.2 (7.3-12.4)	0.141
SNR liver vein	11.2 (10.6-11.9)	12.5 (10.2-13.7)	1.000	11.2 (10.6-11.9)	13.3 (11.9-16.4)	1.000	12.5 (10.2-13.7)	13.3 (11.9-16.4)	0.070
CNR liver vein	8.4 (7.7-8.8)	8.9 (6.2-10.6)	1.000	8.4 (7.7-8.8)	10.4 (8.4-11.5)	1.000	8.9 (6.2-10.6)	10.4 (8.4-11.5)	0.281
SNR right LL	6.9 (6.2-7.7)	7.6 (6.7-9.2)	0.492	6.9 (6.2-7.7)	7.3 (6.4-8.4)	1.000	7.6 (6.7-9.2)	7.3 (6.4-8.4)	0.176
CNR right LL	3.7 (2.9-4.3)	3.9 (3.2-4.6)	1.000	3.7 (2.9-4.3)	4.3 (3.3-4.9)	1.000	3.9 (3.2-4.6)	4.3 (3.3-4.9)	1.000
SNR left LL	6.6 (5.9-7.2)	6.6 (6.4-8.8)	1.000	6.6 (5.9-7.2)	6.4 (6.2-8.4)	1.000	6.6 (6.4-8.8)	6.4 (6.2-8.4)	0.035
CNR left LL	3.7 (2.9-4.2)	3.8 (2.9-4.5)	1.000	3.7 (2.9-4.2)	4.0 (3.2-4.8)	1.000	3.8 (2.9-4.5)	4.0 (3.2-4.8)	0.141
SNR spleen	8.7 (7.2-11.3)	9.0 (7.6-9.6)	1.000	8.7 (7.2-11.3)	9.0 (7.7-9.6)	1.000	9.0 (7.6-9.6)	9.0 (7.7-9.6)	1.000
CNR spleen	6.2 (4.9-7.7)	4.9 (3.3-5.5)	0.703	6.2 (4.9-7.7)	5.5 (4.0-6.2)	1.000	4.9 (3.3-5.5)	5.5 (4.0-6.2)	0.141
SNR kidney	11.4 (10.2-16.9)	12.1 (10.7-14.6)	1.000	11.4 (10.2-16.9)	13.1 (11.9-13.9)	1.000	12.1 (10.7-14.6)	13.1 (11.9-13.9)	1.000
CNR kidney	8.3 (8.1-13.5)	8.3 (7.2-9.9)	1.000	8.3 (8.1-13.5)	9.9 (8.4-10.6)	1.000	8.3 (7.2-9.9)	9.9 (8.4-10.6)	0.070

Data shown as median [interquartile range], SNR = signal-to-noise ratio, CNR = contrast-to-noise ratio, LL = liver lobe. P-value shown after Bonferroni correction

Supplementary Table S4 Signal-to-noise ratio, Contrast-to-noise ratio - subgroup (with EID-CT_{120ml}, and same kVp (120), n = 6)

	**EID-CT, 120 ml**	**PCD-CT 120 ml, 70 keV**	**P-Value**	**EID-CT, 120 ml**	**PCD-CT 100 ml, 70 keV**	**P-Value**	**EID-CT, 120 ml**	**PCD-CT 100 ml, 60 keV**	**P-Value**
SNR asc. Aorta	13.5 (10.8-18.2)	17.3 (12.9-18.8)	1.000	13.5 (10.8-18.2)	16.3 (15.0-17.4)	1.000	13.5 (10.8-18.2)	16.9 (15.6-18.2)	1.000
CNR asc. Aorta	10.4 (6.7-12.1)	12.6 (6.3-12.7)	1.000	10.4 (6.7-12.1)	9.0 (8.4-12.2)	1.000	10.4 (6.7-12.1)	10.8 (10.0-13.4)	1.000
SNR desc. Aorta	12.8 (10.1-14.1)	15.8 (13.1-18.6)	1.000	12.8 (10.1-14.1)	15.6 (13.4-16.3)	1.000	12.8 (10.1-14.1)	15.6 (13.8-17.6)	1.000
CNR desc. Aorta	8.4 (7.9-9.4)	11.5 (6.5-13.5)	1.000	8.4 (7.9-9.4)	9.3 (6.5-11.3)	1.000	8.4 (7.9-9.4)	11.1 (7.9-9.4)	1.000
SNR abd. Aorta	8.2 (7.8-8.3)	10.6 (8.2-11.1)	1.000	8.2 (7.8-8.3)	10.4 (7.8-11.6)	1.000	8.2 (7.8-8.3)	10.4 (8.3-13.0)	1.000
CNR abd. Aorta	5.0 (4.5-5.1)	7.9 (4.8-8.1)	1.000	5.0 (4.5-5.1)	5.5 (3.9-8.0)	1.000	5.0 (4.5-5.1)	5.6 (5.2-9.4)	1.000
SNR portal vein	8.9 (8.2-11.1)	11.8 (10.2-13.4)	1.000	8.9 (8.2-11.1)	11.2 (11.0-13.2)	1.000	8.9 (8.2-11.1)	12.3 (11.8-14.8)	1.000
CNR portal vein	6.9 (5.9-8.3)	9.4 (7.5-10.3)	1.000	6.9 (5.9-8.3)	8.9 (6.9-10.8)	1.000	6.9 (5.9-8.3)	10.5 (8.3-12.5)	1.000
SNR liver veins	9.1 (8.6-9.6)	11.4 (10.6-11.5)	1.000	9.1 (8.6-9.6)	12.7 (12.3-12.8)	1.000	9.1 (8.6-9.6)	12.9 (12.1-14.7)	1.000
CNR liver veins	5.9 (5.4-6.4)	7.8 (6.7-8.3)	1.000	5.9 (5.4-6.4)	8.4 (7.3-9.1)	1.000	5.9 (5.4-6.4)	9.5 (8.4-10.9)	1.000
SNR right LL	5.0 (4.9-5.7)	5.9 (4.6-6.8)	1.000	5.0 (4.9-5.7)	6.7 (5.7-6.8)	1.000	5.0 (4.9-5.7)	6.3 (5.7-6.5)	1.000
CNR right LL	2.5 (1.6-3.1)	2.2 (1.9-3.2)	1.000	2.5 (1.6-3.1)	2.6 (2.2-3.2)	1.000	2.5 (1.6-3.1)	2.7 (2.3-3.5)	1.000
SNR left LL	5.2 (4.9-6.0)	5.7 (4.8-6.0)	1.000	5.2 (4.9-6.0)	6.6 (6.0-7.1)	1.000	5.2 (4.9-6.0)	6.4 (5.4-6.5)	1.000
CNR left LL	2.0 (1.6-3.1)	2.3 (2.0-2.9)	1.000	2.0 (1.6-3.1)	2.9 (2.3-3.5)	1.000	2.0 (1.6-3.1)	3.2 (2.2-3.5)	1.000
SNR spleen	6.9 (6.3-7.3)	7.9 (7.3-10.0)	1.000	6.9 (6.3-7.3)	8.1 (7.5-8.6)	1.000	6.9 (6.3-7.3)	7.7 (7.1-8.6)	1.000
CNR spleen	3.7 (3.2-4.4)	5.2 (5.1-7.5)	1.000	3.7 (3.2-4.4)	4.5 (3.6-5.4)	1.000	3.7 (3.2-4.4)	4.9 (4.0-6.1)	1.000
SNR kidney	11.0 (10.1-11.7)	11.1 (10.1-12.4)	1.000	11.0 (10.1-11.7)	12.5 (9.3-13.2)	1.000	11.0 (10.1-11.7)	12.9 (9.2-13.4)	1.000
CNR kidney	7.2 (6.5-8.6)	8.3 (8.0-9.8)	1.000	7.2 (6.5-8.6)	8.9 (7.2-9.5)	1.000	7.2 (6.5-8.6)	10.0 (8.4-10.6)	1.000

Data shown as median

[interquartile range], SNR = signal-to-noise ratio, CNR = contrast- to-noise ratio, LL = liver lobe

Supplementary Table S5 Correlation between quantitative and qualitative assessment

	Rater 1			Rater 2		
	**PCD-CT 120 ml, 70 keV**	**PCD-CT 100 ml, 70 keV**	**PCD-CT 100 ml, 60 keV**	**PCD-CT 120 ml, 70 keV**	**PCD-CT 100 ml, 70 keV**	**PCD-CT 100 ml, 60 keV**
CNR asc. Aorta	Rho= -0.560<br>P= 0.256	Rho= -0.130<br>P= 1.000	*Rho= -0.448*<br>*P= 0.012*	Rho= -0.296<br>P= 0.388	Rho= -0.164<br>P= 1.000	Rho= -0.370<br>P= 0.087
CNR desc. Aorta	*Rho= -0.498*<br>*P= 0.003*	Rho= -0.148<br>P= 1.000	*Rho= -0.494*<br>*P= 0.003*	Rho= -0.251<br>P= 0.803	Rho= -0.064<br>P= 1.000	*Rho= -0.401*<br>*P= 0.042*
CNR abd. Aorta	*Rho= -0.664*<br>*P= 0.041*	Rho= -0.130<br>P= 1.000	Rho= -0.391<br>P= 0.059	*Rho= -0.436*<br>*P= 0.022*	Rho= -0.147<br>P= 1.000	Rho= -0.278<br>P= 0.523
CNR portal vein	Rho= -0.599<br>P= 0.066	Rho= -0.229<br>P= 1.000	Rho= -0.543<br>P= 0.549	*Rho= -0.438*<br>*P= 0.021*	Rho= -0.086<br>P= 1.000	*Rho= 0.433*<br>*P= 0.024*
CNR liver veins	Rho= -0.251<br>P= 1.000	Rho= -0.076<br>P= 1.000	Rho= -0.308<br>P= 0.477	Rho= -0.009<br>P= 1.000	Rho= -0.171<br>P= 1.000	Rho= -0.267<br>P= 0.861
CNR right LL	*Rho= -0.407*<br>*P= 0.050*	Rho= -0.041<br>P= 1.000	Rho= -0.293<br>P= 0.436	Rho= -0.139<br>P= 1.000	Rho= -0.071<br>P= 1.000	Rho= -0.317<br>P= 0.288
CNR left LL	Rho= -0.429<br>P= 0.033	Rho= 0.0003<br>P= 1.000	Rho= -0.257<br>P= 0.798	Rho= -0.201<br>P= 1.000	Rho= -0.049<br>P= 1.000	Rho= -0.325<br>P= 0.264
CNR spleen	*Rho= -0.644*<br>*P= 0.040*	Rho= -0.246<br>P= 0.899	*Rho= -0.638*<br>*P= 0.042*	Rho= -0.556<br>P= 0.442	Rho= -0.062<br>P= 1.000	*Rho= -0.484*<br>*P= 0.006*
CNR kidney	Rho= -0.596<br>P= 0.075	Rho= -0.184<br>P= 1.000	*Rho= -0.684*<br>*P< 0.001*	Rho= -0.624<br>P= 0.081	Rho= -0.124<br>P= 1.000	Rho= -0.604<br>P= 0.153

Spearman correlations between subjective analysis (Likert scale) and quantitative measurement (CNR). Rho shown for different regions separately for both raters. P-values shown after Bonferroni correction. Significant results shown in *Italics*

Supplementary Fig. S1: Boxplots for the assessment of abdominal organ CNR (contrast-to-noise ratio) within the subgroup (with EID-CT_{120ml}, n=33)

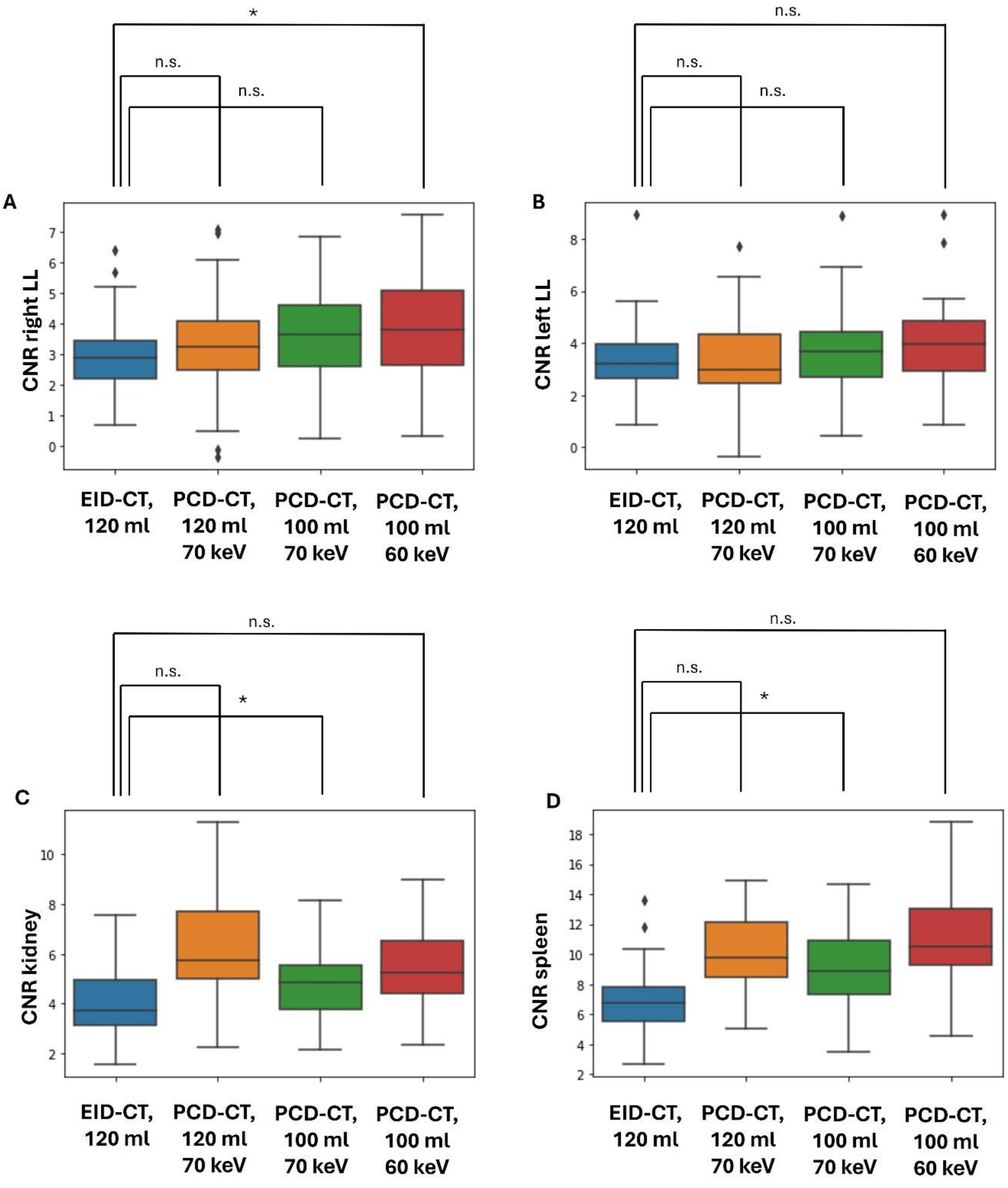

Supplementary Fig. S2: Stacked bar charts showing the distribution of ratings given for overall image quality and overall contrast enhancement within the subgroup (with EID-CT; n = 33).

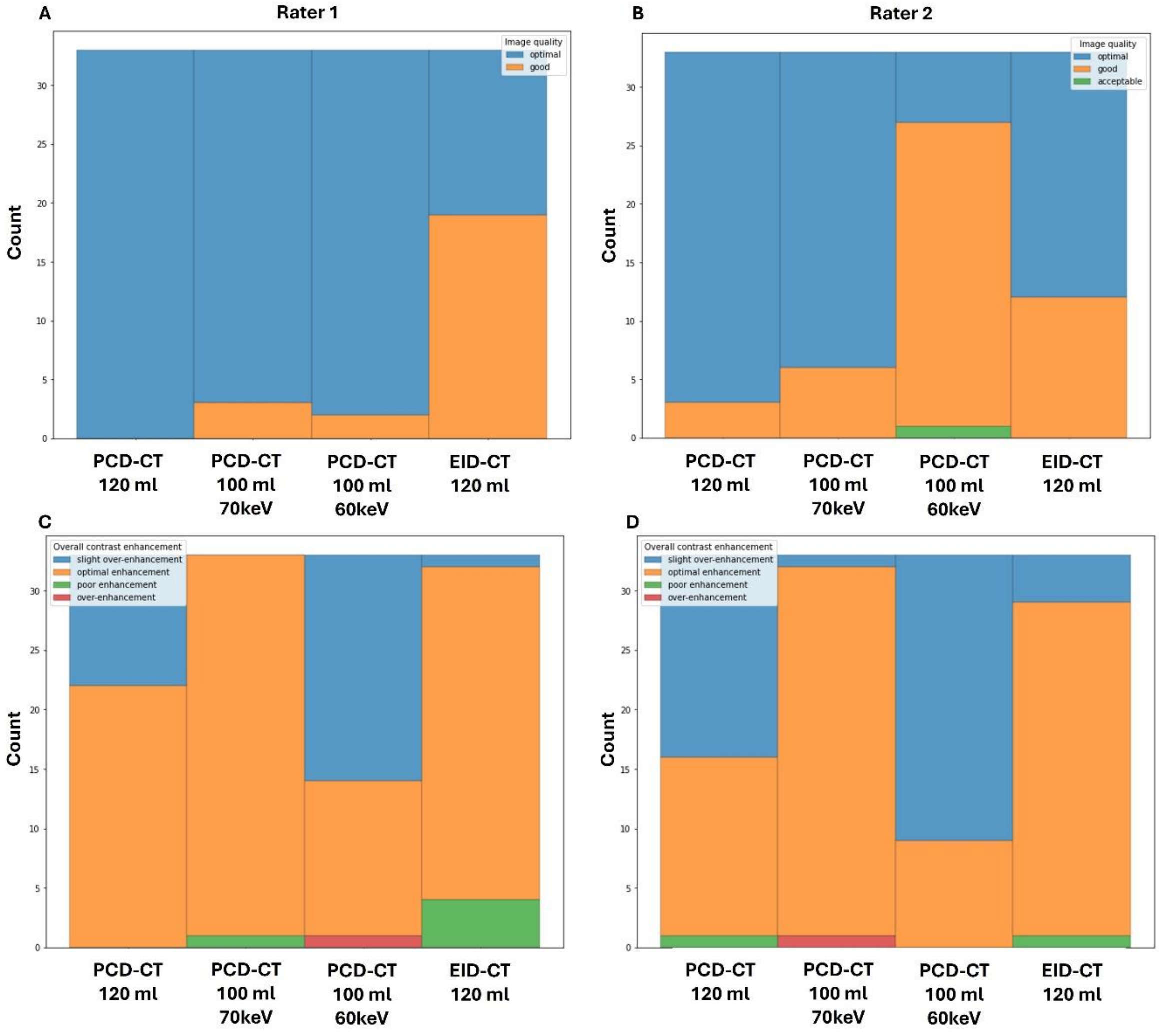

Supplement: Supplementary file 1 — Electronic Supplementary Material [file 330_2025_11409_MOESM1_ESM.pdf]
